# Statistical Mapping of Maize Bundle Intensity at the Stem Scale Using Spatial Normalisation of Replicated Images

**DOI:** 10.1371/journal.pone.0090673

**Published:** 2014-03-12

**Authors:** David Legland, Marie-Françoise Devaux, Fabienne Guillon

**Affiliations:** 1 INRA, UMR782 Food Process Engineering and Microbiology, Thiverval-Grignon, France; 2 AgroParisTech, UMR782 Food Process Engineering and Microbiology, Thiverval-Grignon, France; 3 INRA, UMR 1318 Institut Jean-Pierre Bourgin, Versailles, France; 4 AgroParisTech, Institut Jean-Pierre Bourgin, Versailles, France; 5 INRA, UR1268 Biopolymers, Interactions and Assemblies, Nantes, France; Centrum Wiskunde & Informatica (CWI) & Netherlands Institute for Systems Biology, Netherlands

## Abstract

The cellular structure of plant tissues is a key parameter for determining their properties. While the morphology of cells can easily be described, few studies focus on the spatial distribution of different types of tissues within an organ. As plants have various shapes and sizes, the integration of several individuals for statistical analysis of tissues distribution is a difficult problem. The aim of this study is to propose a method that quantifies the average spatial organisation of vascular bundles within maize stems, by integrating information from replicated images. In order to compare observations made on stems of different sizes and shapes, a spatial normalisation strategy was used. A model of average stem contour was computed from the digitisation of several stem slab images. Point patterns obtained from individual stem slices were projected onto the average stem to normalise them. Group-wise analysis of the spatial distribution of vascular bundles was applied on normalised data through the construction of average intensity maps. A quantitative description of average bundle organisation was obtained, via a 3D model of bundle distribution within a typical maize internode. The proposed method is generic and could easily be extended to other plant organs or organisms.

## Introduction

Crop species like maize (*Zea mays L.*) are of increasing interest for cattle feeding or for bioethanol production [1]–[3], and many studies have been devoted to the elucidation of relationships between cell wall chemical composition and degradability [4]–[6]. In addition to the variability of the composition of cell types, the morphology of cells should be taken into account to better understand plant degradation or mechanical properties [7], [8]. Cell morphology is typically investigated through the distribution of morphometric features that quantify cell size and shape [9]–[12]. At a larger scale, the proportion and the spatial distribution of tissues within plant organs may impact the mechanical and degradation properties of the tissues. Few attempts have been made to obtain a quantitative description of this spatial organisation.

The general aim of this work was to develop generic procedures to establish statistical 3D mappings of biological structures organisation within an organ. Maize internodes are taken as a model of the lignocellulosic stem. In the present study, the spatial organisation of vascular bundles within the stem is considered. Vascular bundles are important in terms of stem structure and development. They are composed of several types of cells with different compositions and morphologies [13], [14]. It is suspected that they have a strong impact on the mechanical behaviour of the whole stem. As they are hardly degradated by enzymatic reactions, they directly impact the global degradability of the stem. In addition, the size, the shape and the orientation of the parenchyma cells vary with the distance to the nearest bundle. Vascular bundles do not form a continuous tissue, but may be instead considered as individual objects within the stem cross-section. We propose to describe their spatial organisation using tools from spatial statistics. Within this framework, the observed point patterns are considered as random realisations of a more general point process [15]–[17]. The aim is to describe the process on the basis of measurements made on one or several representative observations. The simplest tool to describe a point process is the intensity, which corresponds to the expectation of the number of points within a given region. More complicated tools can be used to describe interactions between points, such as repulsion or clustering. In the present work, the spatial distribution of vascular bundles is investigated through the construction of average intensity maps, which integrate observations made on several samples.

Due to experimental factors and to biological variability, stems exhibit a strong heterogeneity in shape and size, making it difficult to compare or summarise individual observations. A possible solution is to consider a reference structure, such as the organ boundary, and study variations of tissue morphology as a function of the distance to the boundary. This results in morphology profiles, that can be normalised for allowing comparison between individuals [18]–[20]. In order to analyse differences in spatial organisation, reduction to profiles is not sufficient. A better approach is to apply a geometric transform that project each individual observation into the same reference shape. This type of question is very common in medical image analysis when an individual human brain has to be compared to a reference atlas, or for building the reference atlas by merging a collection of individual images [21], [22]. Such questions also arise at lower scales, for describing spatial organisation within a reference cell or organ [23], [24]. An underlying assumption in cartography or atlas construction is the homology of the structures, i.e., the direct correspondence of pixels or voxels belonging to different subjects. In the case of plant stems, the number and the position of the cells and of the vascular bundles vary between plants, i.e., homology cannot be easily found. For the contour of the organ, some homology can generally be assumed. Each contour may be deformed to match a reference contour, and it is possible to merge several images. The reference contour can be explicitly chosen from the set of individual contours. A better approach is to compute an average contour that summarises the set of contours. Several families of methods have been developed for modelling shape contours. Euclidean Fourier descriptors are quite popular for identifying the most relevant variations in shape, but their interpretation can be difficult [25]–[28]. Statistical analysis of shapes is a more general approach that describes structures by a set of landmarks located at particular positions, typically points of high curvature [29]–[31]. For both approaches, appropriate statistical tools make it possible to extract the average shape as well as the variations of shapes around the average.

In order to build a statistical map, data from replicated images acquired for several plants are integrated. The vascular bundles correspond to observed point patterns that are bounded by the stem contour and that are repeated over several stems and positions within the stem. This study aims at estimating the intensity map of vascular bundles based on the replicated data. In order to compare intensity maps for different experimental factors, it is necessary to build a reference stem model. The reference stem is obtained by modelling the contour of several stem slabs. The positions of the vascular bundles are obtained by macroscopy imaging and projected onto the reference stem mode. Spatial normalisation makes it possible to compute an intensity map of vascular bundles averaged over several stem images.

## Materials and Methods

### Material

**Figure 1:**
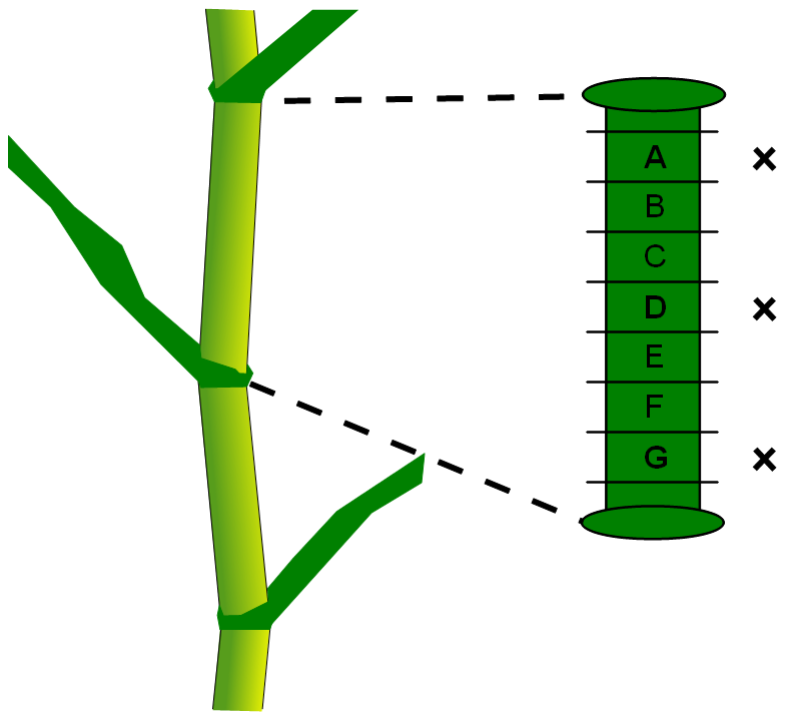
Sampling of a maize internode. All slabs were used for contour modelling. Only slabs A, D and G (marked with a cross) of each internode were used for macroscopy imaging.

Maize internodes were provided by Biogemma (Paris, France), within the project GRASSBIOFUEL. Two genotypes were considered: the wild type, and a mutation on a gene coding for an UDP D-glucose dehydrogenate [32], [33]. Seven stems were sampled from each genotype. For each stem, the internode located below the first ear was truncated into seven or eight 1 cm high slabs. Slabs were numbered from A to G or H, starting from the top of the internode (Fig. 1).

### General approach

**Figure 2:**
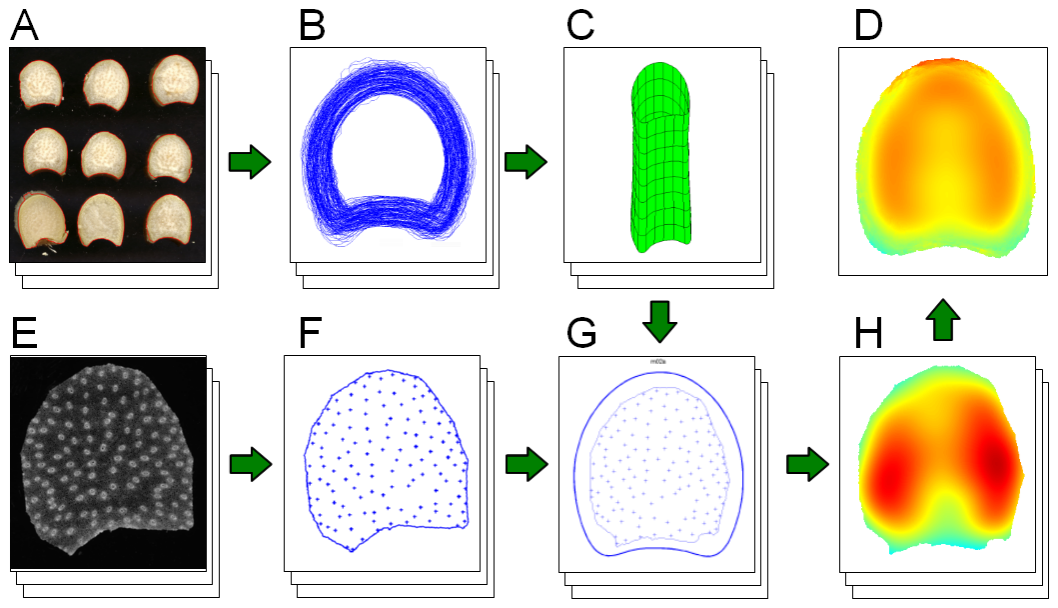
Graphical abstract of the proposed methodology. (A) Image acquisition of maize stem slabs. (B) Spatial normalisation and alignment of slab contours. (C) Modelling of a reference stem. (D) Global intensity map obtained by averaging individual normalised maps. (E) Acquisition of macroscopy image of stem sections. (F) Automatic segmentation of vascular bundles. (G) Projection of bundle positions obtained in F onto the reference space, using the stem model obtained in C. (H) Computation of intensity map for the sample image projected onto the reference space.

Figure 2 gives a graphical overview of the overall proposed methodology. The first step was to define a common reference to compare all observations. The reference stem was obtained by computing a statistical model of the average stem contour. For practical reasons, it was necessary to remove the bark of the stems before slicing to obtain vascular bundle observations. It was therefore not possible to use the same images for computing the average stem contour and for identifying the position of vascular bundles. Scan images of stem slabs acquired before bark removal were used for computing the contour of the reference stem (Figs. 2-A,B,C). Macroscopic images of stem slices after bark removal were used for identifying the position of individual vascular bundles (Fig. 2-E). A spatial normalisation procedure is applied to the positions of the vascular bundles in each stem slice, resulting in a set of points projected onto the reference stem (Figs. 2-F,G). Once projected onto a common reference space, the normalised point patterns can be described through their intensity map (Fig. 2-H). Since intensity maps are computed in the same reference space, it is possible to compute an average intensity map that takes all of the images into account. The result is a representation of the vascular bundle intensity variations within a reference stem (Fig. 2-D).

### Modelling the 3D stem shape

#### Images of slab contours

**Figure 3:**
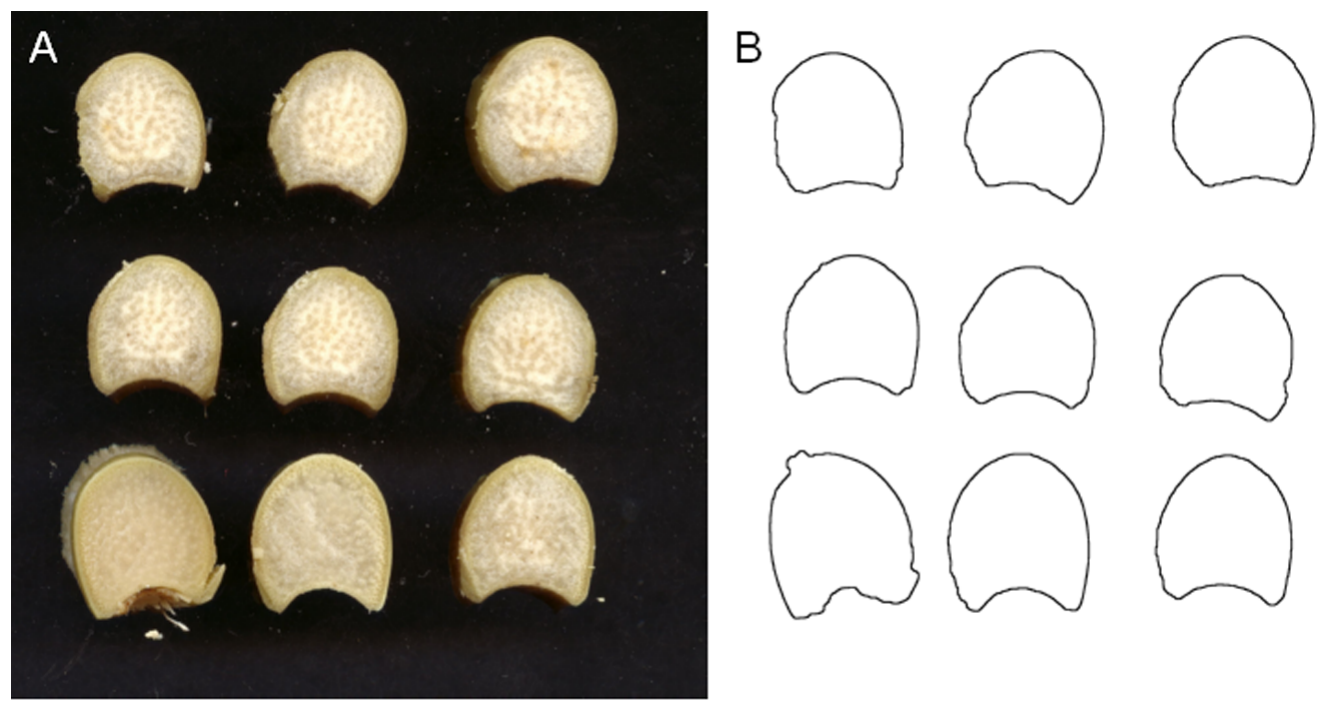
Imaging of slab contours. (A) Acquisition of slab images for a sample internode. (B) Automatic segmentation of slab contours.

Colour images of stem slabs were acquired using a flat scanner. Each side of the slabs was observed, resulting in a symmetric replicated observation for each slab section. The red, green and blue channels were coded with values between 0 and 255. Images were acquired with a resolution of 720DPI, corresponding to 35.3 µm/pixel (Fig. 3-A).

Slab images were converted to Hue-Saturation-Value colour space, and the “value” channel was used for the segmentation of slabs. A threshold was applied to the “value” channel to identify regions corresponding to each slab [34]. The value channel ranged from 0 to 1. The “value” histogram of each image exhibited two peaks around 0.1, corresponding to the background, and 0.8, corresponding to the slabs. A threshold value equal to 0.5 was empirically chosen in between. A binary image of the contour was obtained for each slab face by keeping only boundary pixels (Fig. 3-B), resulting in approximately 14 or 16 contours for each stem.

#### Descriptors of slab contours

**Figure 4:**
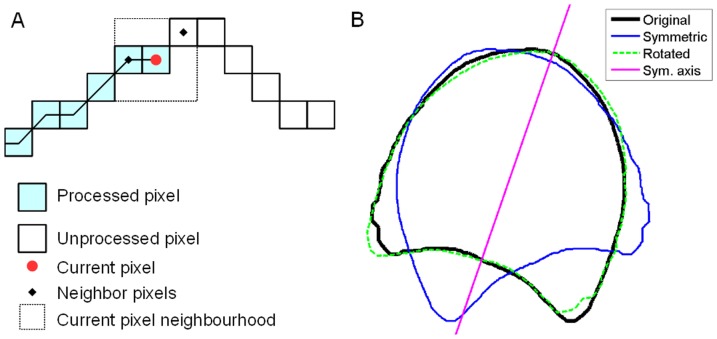
Construction of contour polygons. (A) Chaining contour pixels. (B) Determination of the polygon orientation. Black: original polygon; blue: left-right flipped polygon; green: minimal difference between the two polygons. Pink line: symmetry axis.

In order to compute statistical models of stem shape, descriptors of individual contours that can be compared and averaged must be assessed. The contours were described as polygons rotated to have the same orientation and the same number of vertices whose coordinates are expressed in relation to to the centre of gravity of the slab. The procedure consisted first in chaining the adjacent pixels of the contour (Fig. 4-A). To ensure that contour width was one pixel, a homotopic thinning was first applied to the binary image [35]. An initial pixel was arbitrarily chosen on the contour. The neighbours of the current pixel were considered, and the first unprocessed neighbour was chosen as the next current pixel. The polygon was obtained by iterating along the pixels of the contour until coming back to the initial pixel. The resulting polygons were translated so that their centre of gravity would be located at the origin and eventually re-oriented so that their orientation would be counter-clockwise.

Polygons were rotated so that their symmetry axis would be aligned with the vertical axis. The symmetry axis of each polygon was identified by flipping the polygon around the *y*-axis and rotating the flipped polygon so that the difference between the two polygons was minimal (Fig. 4-B). The difference between the two polygons was obtained by computing the sum, over all vertices of rotating polygon, of the minimal distance between current vertex and the original polygon. The minimum was computed using golden section search and parabolic interpolation, by calling the “fminbnd” Matlab's function. The rotation angle minimizing the difference was divided by 2, and the corresponding rotation transform was applied to the original polygon, resulting in a polygon pointing upwards. The vertex located on top of each polygon was chosen as initial vertex (i.e. with index equal to 1). The vertex number of each contour polygon was reduced to the same number by sub-sampling, starting from the top vertex. The number *n*_v_ of vertices of resampled polygons was chosen equal to 200. The position of each vertex was defined by the two *x* and *y* coordinates expressed in relation to the centre of gravity. Each contour was therefore described by the *n*_c_ = 2×*n*_v_ = 400 coordinates of the vertices of its polygon. All the resulting polygons started from the upper vertex, had the same number of vertices, and were oriented counter-clockwise. The vertices of all polygons were assumed to be in direct correspondence. A data table *XY*(*i*, *j*) was built with the rows *i* corresponding to the individual slabs and the columns *j* corresponding to the 400 *x* and *y* coordinates of the polygon vertices.

#### Statistical analysis of stem contours

Statistical analysis of the contour was done in two steps. First, a principal components analysis was applied to help identifying the slab populations with similar or different contours. Then an analysis of variance was applied on the first principal components to identify which factors were relevant for modelling.

Principal components analysis was applied on the *XY* data table formed by the coordinates of slab contours. Principal component analysis is a multidimensional data treatment that reveals the similarities between samples by taking all variables into account. Similarity maps, drawn from the principal component scores, are used to compare the samples and to identify clusters of similar samples. Applied to ordered signals such as polygon coordinates, synthetic polygons can be reconstructed from principal component loadings, highlighting changes from the average contour.

A general linear model was applied to each of the first five principal component scores *SC*(*i*, *j*), *i* being the index of the slab contour, and *j* the principal component index. The general linear model used in this study took into account the fixed effect *α_G_* of the genotype *G* (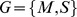), *β_C_* the fixed effect of the cutting position *C* (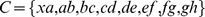, “*ab*” being the cutting position between slabs A and B), and the fixed effect 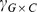 of their interaction. A random effect 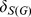 of the stem *S* nested in the genotype *G* was taken into account, resulting in the final model:
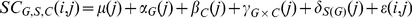
(1)where *μ*(*j*) is the intercept and *ε*(*i*, *j*) is the residual error. An analysis of variance was applied to each of the effects and for each of the first five principal components. The effects whose p-value were greater than 0.05 were considered as not signifiant and were not taken into account for the statistical modelling.

#### Statistical modelling of stem contours

The aim of contour modelling was (1) to define a reference stem contour used to project all observations and (2) to compute an estimate of the stem contour corresponding to each macroscopy image. A simplified general linear model was computed on the data table *X*(*i*, *j*) corresponding to the vertex coordinates. Removing non relevant effects improves the estimation of the other effects. The linear model took into account the fixed effects of the genotype, of the cutting position, and the random effect of the stem nested to the genotype:
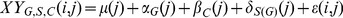
(2)


The intercept values *μ*(*j*) were used to compute the reference average contour that will be used for subsequent spatial normalisation. The estimated coefficients 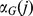 and 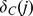 were used together with the intercepts to compute the model contour for each genotype and for each cutting position respectively. Combining the genotype and the cutting position coefficients made it possible to reconstruct 3D models of internodes for each genotype.

The slab effects were estimated for each of the three slabs *A*, *D* and *G* that were sampled for macroscopy imaging. The estimated coefficients 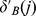, 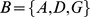 were obtained from the average of coefficients for the two cutting positions around each slab. The stem contour corresponding to each macroscopy image was obtained by adding the intercepts *μ*(*j*) with the genotype, slab and stem effects corresponding to the image.

### Estimation of vascular bundle intensity

#### Imaging vascular bundles

**Figure 5:**
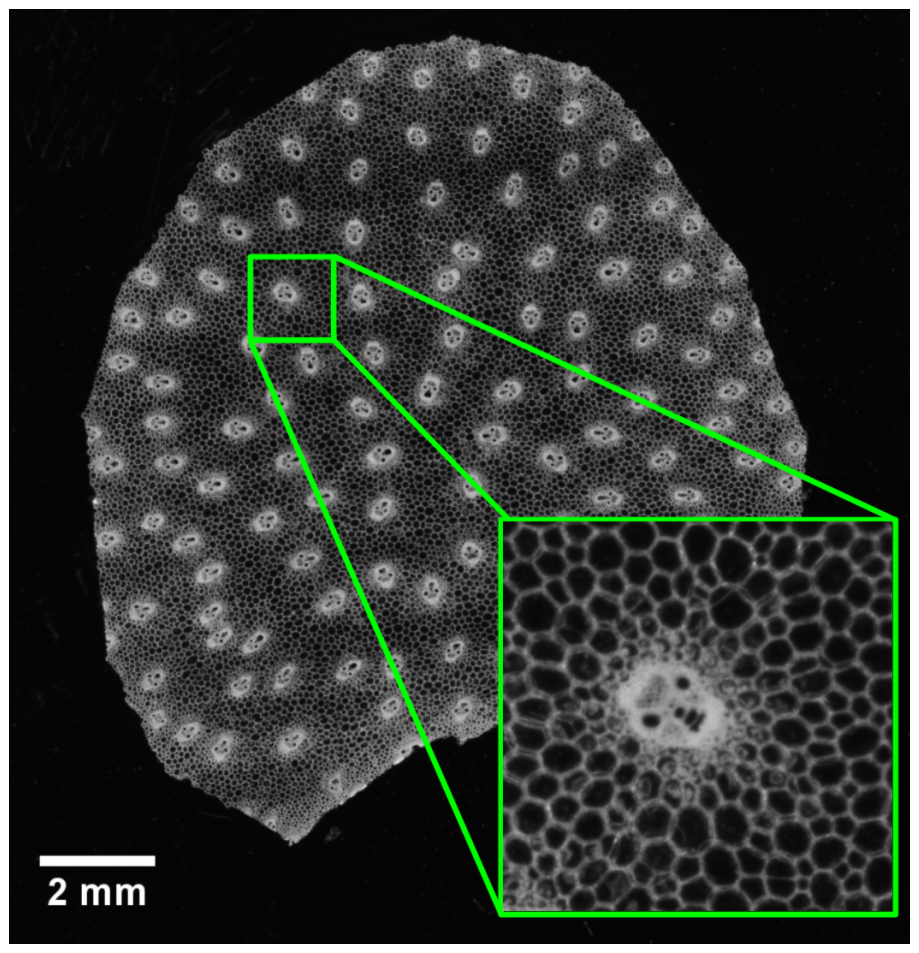
Imaging of vascular bundles. Sample image of stem section obtained by macroscopic imaging, with a detail of the whole image.

Macroscopy imaging was used to visualise vascular bundles within the whole stem cellular structure. Three slabs were chosen to study bundle intensity in the upper, middle, and lower part of each stem, corresponding to labels A, D and G (Fig. 1). The bark of each slab was manually removed to allow slicing of the whole stem section. Slices (150-µm thick) were obtained using a vibrating lame microtome (MICROM, HM 650 V, Microm International GmbH, Walldrof, Germany). Stem slices were imaged using the macroscopy imaging system developed at INRA Nantes, and known as the “BlueBox” [36], [37]. The device provides dark field images with a field of view of approximately 25 mm^2^ to 1 cm^2^, with a resolution of 3–7 µm. Such images have been shown to be relevant for quantifying the cellular morphology of plant tissues such as tomato pericarp [19], [36] and apple parenchyma [38]. From 12 to 16 images per slice were combined to obtain mosaic images of the whole stem parenchyma sections. Images were stitched by using a multiresolution pyramid approach [39]. The size of mosaic images was approximately 4500×4500 pixels, with a resolution equal to 3.62 µm by pixel, and grey values coded between 0 (black) and 255 (white). As the diameter of most parenchyma cells ranged between 50 and 150, the resolution of macroscopy images made it possible to observe the cellular morphology. Moreover, the field of view gave access to the global organisation of plant tissues within the inner parenchyma of the stem (Fig. 5).

#### Segmentation of slice contours

**Figure 6:**
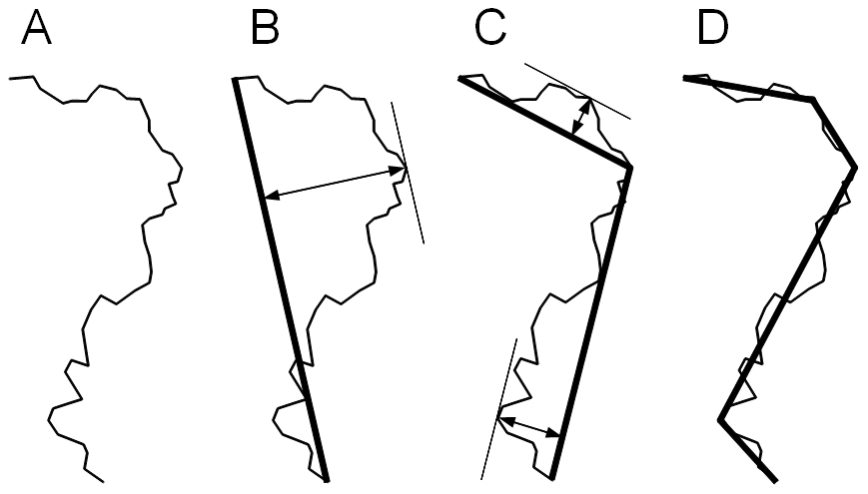
Simplification of a polygonal curve with the Douglas-Peucker algorithm. (A) Original polygonal curve. (B) First approximation by a line segment, and determination of the furthest vertex. (C) Splitting of the curve into two sub-curves, and determination of the furthest vertices in each sub-curve. (D) Iteration of the procedure until the distance from the vertices to the simplified curve is lower than a predefined threshold.

Slice contours were obtained by applying a watershed algorithm that detected cells, and then applying a hole-filling algorithm. A morphological closing of radius 30 was applied to smooth the boundary. The contours of the resulting binary images were transformed into polygons using the pixel-chaining procedure described previously. Coordinates were multiplied by image resolution in order to be expressed in millimetres. In order to smooth the contour and reduce further computation time, the vertex number of the contours was reduced using the Douglas-Peucker algorithm [40]. The principle is to recursively subdivide portions of polygons until the maximum distance between original and simplified polygons is below a given tolerance value (Fig. 6). Portions of polygons were subdivided by determining the furthest vertex from the line joining the extremities of the current portion. A tolerance of 100 microns was used, resulting in polygons with vertex numbers between 10 and 30. Each polygon was translated so that its centroid would be located at the origin.

#### Segmentation of vascular bundles

**Figure 7:**
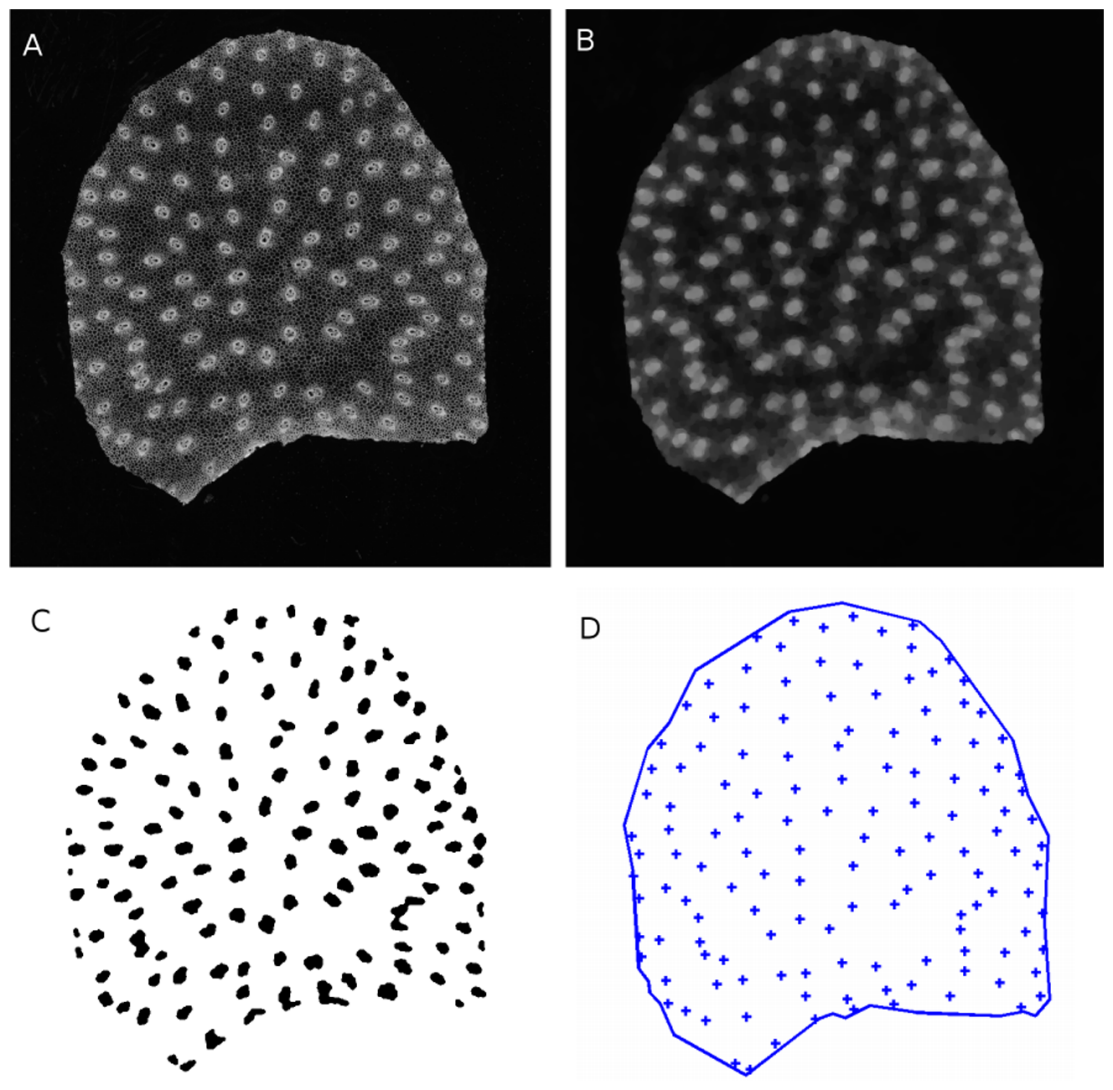
Automatic segmentation of vascular bundles. (A) Original slice image. (B) Result of Alternate Sequential Filtering. (C) Detection of bundles with extended minima. (D) Bundle centroids with corresponding slice contour.

Vascular bundles appeared as sets of small cells with thick walls, with diameters of approximately 300 µm. Since bundles contain several regions with cells of various sizes, differentiating vascular bundles from parenchyma cells is difficult. Images were enhanced by using alternate sequential filters [35], which consist in applying morphological openings and closings of increasing size. Structuring elements were discrete disks with radii ranging from 1 to 10 pixels. This resulted in images without parenchyma cell walls, while vascular bundles were still visible (Fig. 7-B). Vascular bundles were segmented with extended minima filters. A threshold of 30 was used to detect large bundles. A threshold of 20 was used to detect small bundles. To separate collapsing bundles, a watershed was applied to the complement of the distance map (Fig. 7-C). The centroid coordinates of each bundle were computed and translated according to the centroid of the contour. This resulted in a point pattern bounded by the slice contour (Fig. 7-D).

#### Spatial normalisation of macroscopy images

In order to compare stems and vascular bundle intensity, it is necessary to project each individual slice onto the same reference coordinate system by applying a spatial normalisation procedure. The stem contour was used as a reference. One difficulty was that the stem contour was not visible on macroscopy images. Therefore, the spatial normalisation procedure consisted in (1) a rigid transform that replaced the slice contour and the bundles within the individual stem contour obtained from statistical modelling and (2) a non-rigid transform that deformed individual stem contour into the reference stem contour.

**Figure 8:**
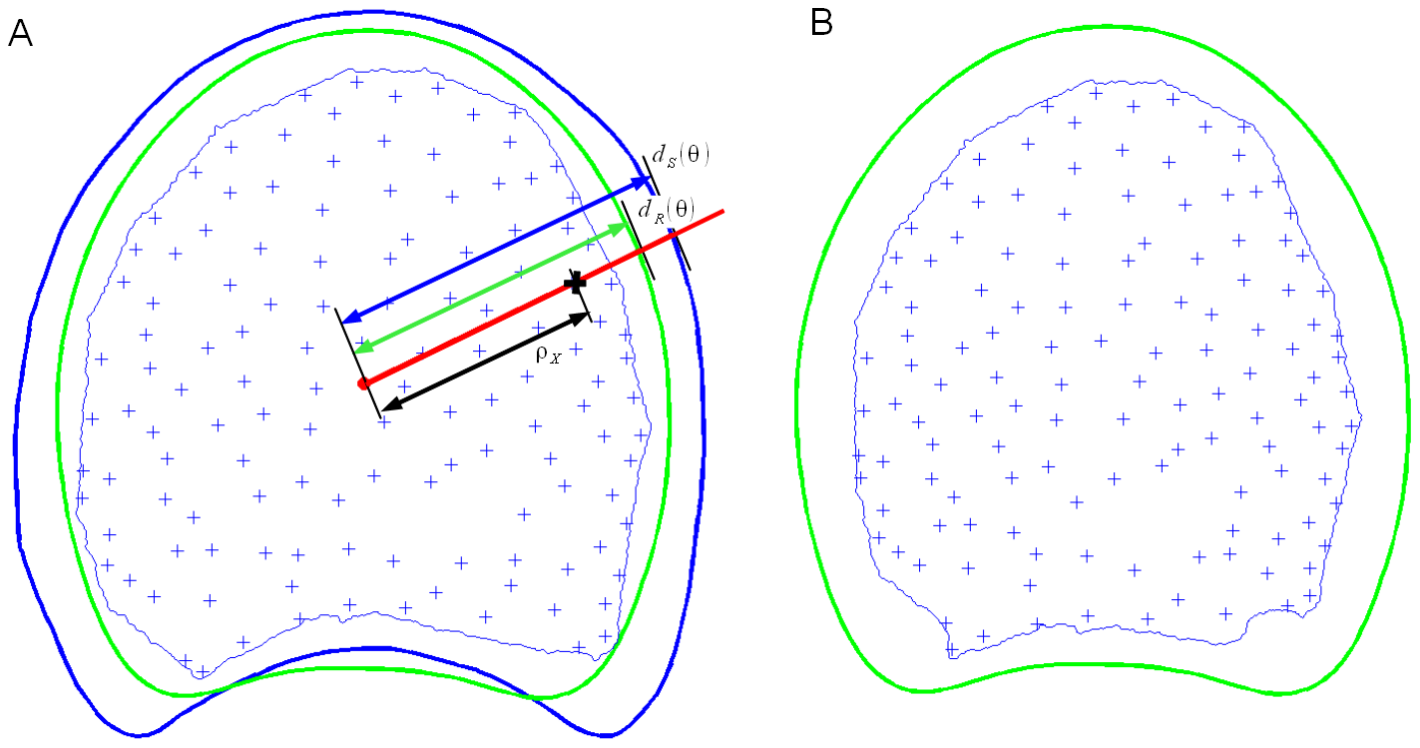
Spatial normalisation of an individual section onto the reference section. (A) Original bundle positions, macroscopy section contour (thin blue), estimated contour of the slab (thick blue), reference stem contour (thick green). (B) Position of bundles and slice contour after spatial normalisation.

The polygons corresponding to the slice contour and the individual stem contour model were superimposed. Both contours were already centered at the origin, but it was necessary to determine the rotation that minimised the difference between the slice contour and the stem contour model. The optimal rotation angle was computed using the procedure described previously for the normalisation of slab contours. The corresponding rotation transform was applied to the coordinates of the slice contour and bundle positions (Fig. 8-A).

The reference stem contour was then considered together with the slice contour and the individual stem contour (Fig. 8-A). Each point within the individual slice was projected into the reference coordinate system using a transform based on polar coordinates. More precisley, the polar coordinates 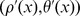 of a point *x* in the reference space were obtained from its polar coordinates 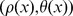 in the space of the individual centred slice. The angular position *θ*(*x*) was maintained, and the distance to origin *ρ*(*x*) was adjusted according to the shape of the stem. For each point *x*, a ray emanating from the origin and passing through the point is considered. The ray intersects both the model contour of the current slice and the reference contour (Fig. 8-A). Distances to the origin of each intersection point are designated 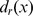 for the reference contour and 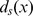 for the contour of the current slice. The normalised coordinates of the point are obtained as the ratio of distances to the origin of the two intersection points (Fig. 8-A):
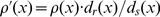
(3)The same normalisation procedure was applied to the position of the bundles and to the vertices of the macroscopy contour. The result was a set of points representing vascular bundles for each slice, together with the contour polygon of the slice, both expressed in the coordinate system of the reference contour (Fig. 8-B).

#### Estimation of vascular bundle densities

Estimation of vascular bundle densities was performed using kernel-based intensity estimation [41], [42]. The principle is to replace each point by a predefined smooth function (called the kernel) centred on the point, and to compute the sum of all shifted kernels. Computations are performed using normalised bundle positions. The contour of each slice was used as a bounding frame. A bundle intensity map was estimated for each individual slice normalised into the reference of the model stem contour, resulting in an estimated map of bundle intensity for each slice. An isotropic gaussian smoothing kernel was used, with a default value calculated by a simple rule of thumb that depended only on the size of the bounding frame. Default edge correction was applied.

#### Software implementation

Image processing was performed within the Matlab environment (The MathWorks, Natick, MA, USA). Geometric operations on polygons and point sets were performed using software developed within the Matlab environment and integrated into “MatGeom”, a freely available library for geometric computing within Matlab (http://matgeom.sourceforge.net/). Estimation of intensity maps was performed within the R Software using the spatstat package [41]. Statistical analyses (principal component analyses, general linear models) were performed within the Matlab environment. In order to facilitate reproducibility of results and their adaptation to other data, the Matlab and R scripts used to process images and geometric data are provided in File S1 and S3. Some pre-processed data files are provided in File S2, and original data are available on request.

## Results

### Statistical analysis and modelling of stems

**Figure 9:**
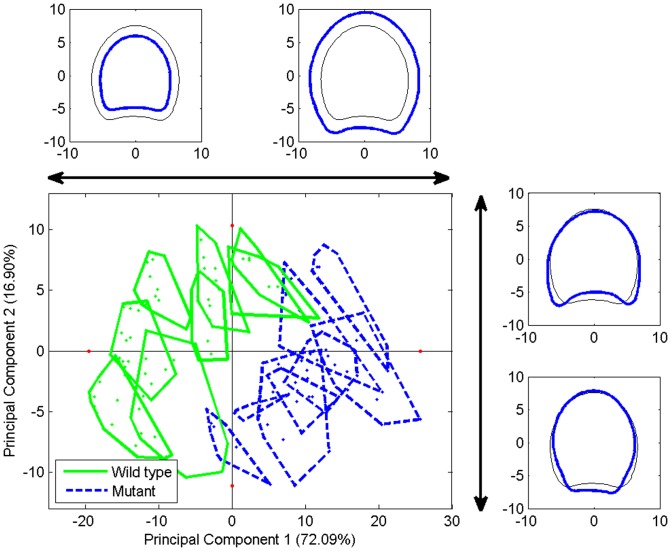
Principal components analysis of stem contours. Similarity map of components 1 and 2. Individuals corresponding to the same stem are represented within a convex polygon. Synthetic profiles corresponding to extreme scores are represented in thick blue together with the reference contour in thin black for component 1 and on the right for component 2.

Figure 9 shows the projection of individual slab contours onto the coordinate system given by the first two principal components. For interpretation, the minimum and maximum scores of each component were used to compute synthetic contours corresponding to the variations described by the component. The first principal component described 72% of the total variance. The corresponding extreme contours were drawn over the similarity map together with the average/reference contour. They clearly show that principal component 1 described the size variations among the slabs. Groups were drawn on the map according to the slab origin for highlighting genotypes and stem effects. Component 1 opposed slab contours of the two genotypes, showing that mutant stem sections were larger than those of the wild type. The second principal component that described 17% of the total variance was interpreted as a difference in shape, revealing a contrast between thick and round contours as opposed to thin and elongated contours. The grouping of individual contours according to the stem was observed. Table 1 presents an analysis of variance performed on the first five principal components. An effect of the genotype was observed for the first principal component, related to the size. In fact, slab sections from the mutant were larger than the wild type. An effect of the cut position was revealed for the first two components. The size of the section varies both in size and in elongation with the position. The stem was also found to have an effect for the five components tested.

**Table 1:** Statistical analysis of contour principal components.

	Inertia	Analysis of variance p-values			
	(%)	genotype	cut	geno×cut	stem
CP1	72.1	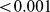	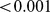	.91	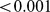
CP2	16.9	.13	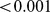	.87	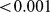
CP3	4.0	.97	.66	.09	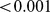
CP4	2.7	.39	.66	.40	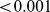
CP5	1.0	.37	.06	.70	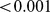

Inertia corresponds to the amount of variability explained by the corresponding component. The analysis of variance results in a p-value computed for each experimental factors of each principal component. Significant p-values (lower than 0.05) are highlighted.

**Figure 10:**
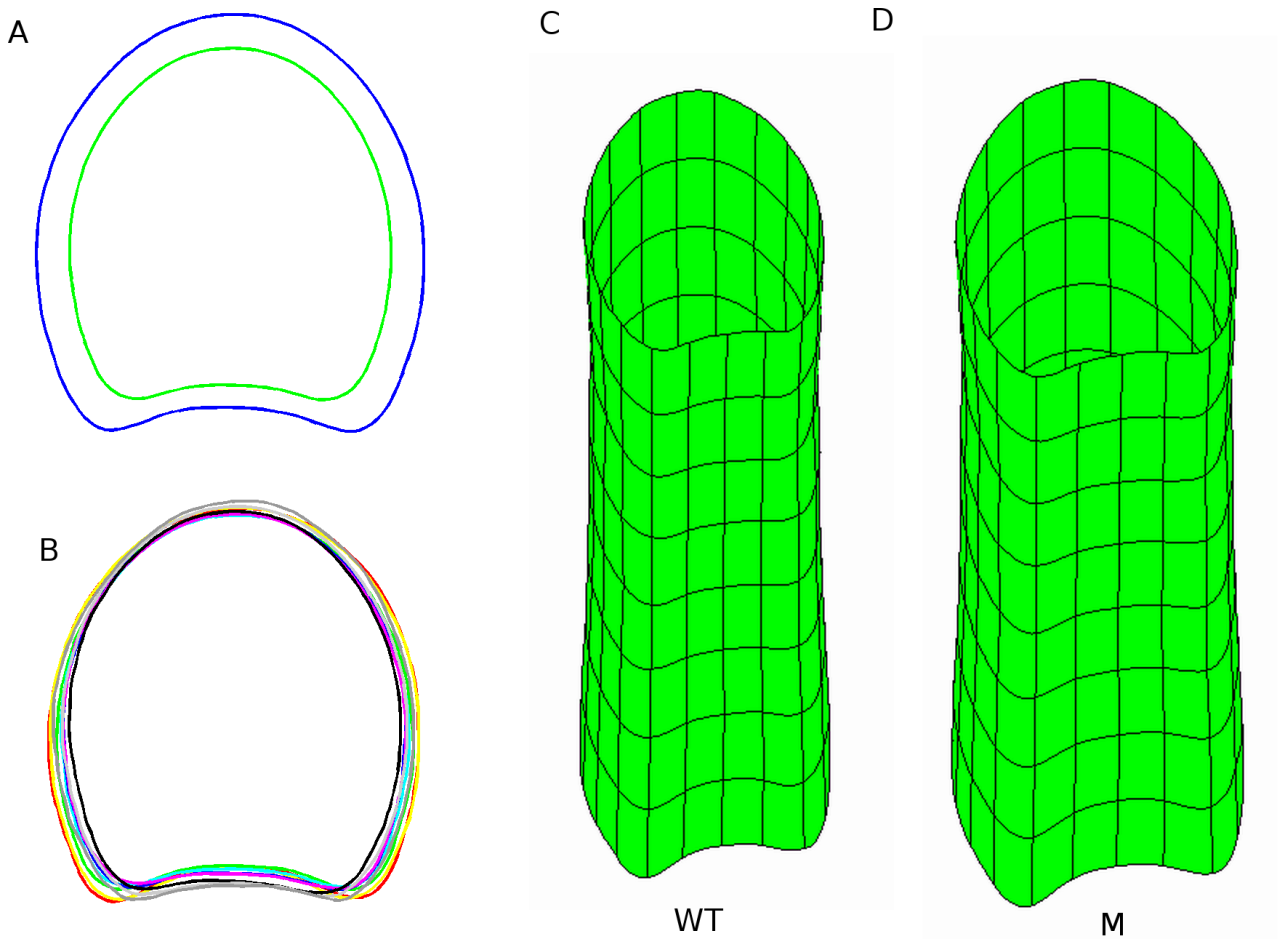
Modelling of slice contours. (A) Average contour of each genotype, exhibiting differences in size. (B) Average contour of each cutting position, exhibiting differences in shape. (C) 3D model of the average wild-type stem. (D) 3D model of the average mutant stem.

The simplified linear model specified in eq. (2) was used to generate reference contours used for spatial normalisation and for graphical representation of results. The genotype stem or cutting position effects made it possible to obtain the average contours of each genotype (Fig. 10-A), stem, and cutting position (Fig. 10-B). The difference in size between genotypes is clearly visible, but no difference in shape could be noticed. On the contrary, the cutting positions showed differences in shapes, but no difference in size. The combination of the genotype with the cutting position effects led to model stems for each genotype (Fig. 10-C, 10-D).

### Spatial normalisation of vascular bundle positions

**Figure 11:**
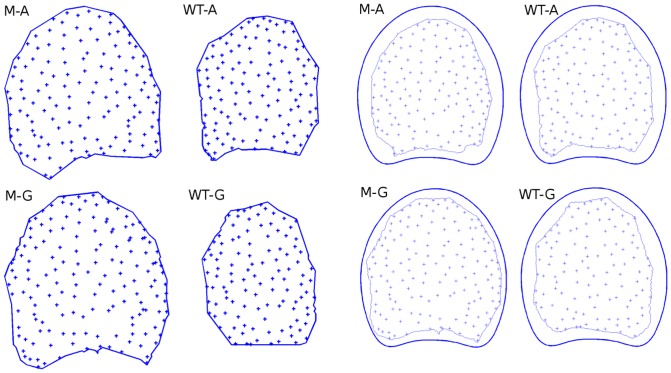
Spatial normalisation of point patterns. Each macroscopic slice is rescaled and deformed to correspond to the reference space. M-A: upper slice of the mutant genotype. M-G: lower slice of the mutant genotype. WT-A: upper slice of the wild type. WT-G: lower slice of the wild type.

Figure 11 shows several point patterns representing vascular bundles before and after spatial normalisation, on four sample slices for the wild type and the mutant genotypes, and for the upper and lower positions. Before spatial normalisation, a regular distribution of bundles could be observed in both genotypes, typical for repulsive point processes. Global intensity was larger in the wild type slices, but no particular variation in local intensity was visible.

Normalisation allows the comparison of the vascular bundle distribution independently of the variations in size and shape of the slabs. Regularity is still visible, but the difference in global intensity is less perceptible due to normalisation.

### Intensity maps of vascular bundles

**Figure 12:**
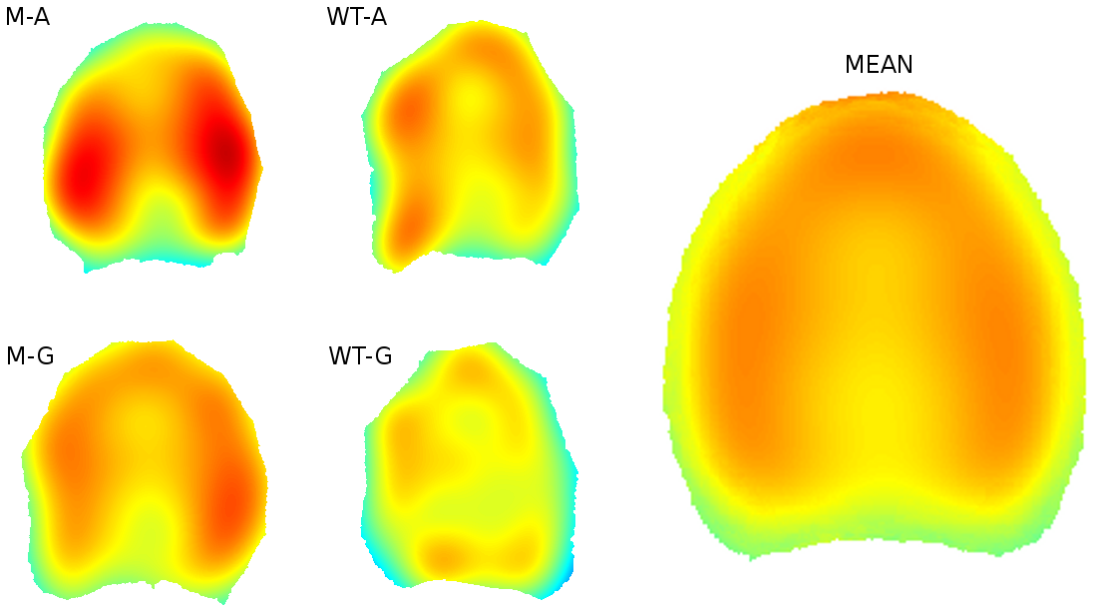
Computation of average intensity map. (A) Individual intensity maps, same as in Fig. 11. (B) Global average intensity map.

Figure 12-A shows bundle intensity maps computed for each normalised slice. Intensity heterogeneity is clearly visible within each individual section: bundles are more numerous on the periphery of the sections and less numerous in the centre. Global intensity was visually greater in the mutant sections. Intensity was estimated for the same positions in each slice, making it possible to superimpose intensity maps. Hence, an average intensity map could be obtained by computing point-wise intensity averages (Fig. 12-B). The average intensity map showed an increase of vascular bundle intensity on the left, right and top parts of the stem section, and a decrease in the centre of the section. Low intensity was observed on the edge of the stem. While edge correction was applied for intensity estimations, intensity maps still seem underestimated at slice edge. Average intensity maps were also computed for each level of the experimental factor, e.g., cutting position, stem and genotype. A higher global intensity of the points was observed for the average intensity map of the mutant genotype. However, no difference in intensity distribution could be observed. A small difference could be observed for average intensity maps estimated for different slabs: bundle intensity is higher for two small lateral regions of the upper slices, for both the wild type and the mutant. This difference in intensity could not be explained, but will be further investigated in forecoming studies.

**Figure 13:**
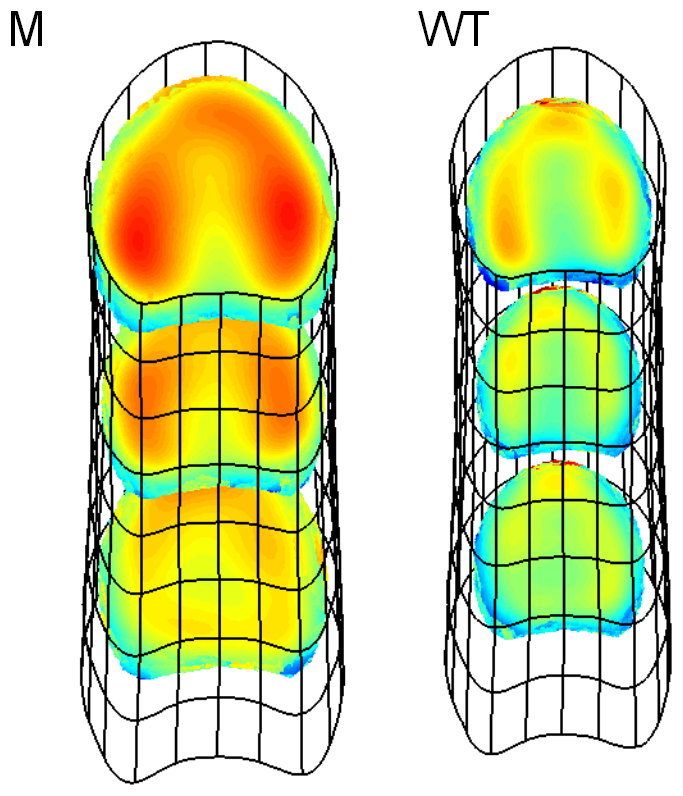
Estimated vascular bundles intensity within model stems. 3D representation of average stem of each genotype, together with estimated intensity map of vascular bundles at three different positions in the stem.

By combining all estimates for the different levels of the experimental factors, it was possible to assess the bundle intensity for different combinations of cutting position and genotype. These average intensity maps are represented together with the corresponding stem models (Fig. 13). A scaling factor was applied to the *x* and *y* coordinates of each map to better fit the corresponding stem model. The stem models show the difference in size of each genotype, as well as the slight variation in size and shape for the different cutting positions. The global difference in intensity between genotypes is clearly visible. For both genotypes, the intensity in the upper sections is globally higher. In all the sections, a contrast is visible between the periphery and the centre.

## Discussion

The spatial organisation of vascular bundles in maize stems was investigated using tools from spatial statistics together with a spatial normalisation procedure. A statistical model of the internode contour was computed using the whole data set, therefore providing a reference space for comparing observations. Vascular bundle positions were projected onto this reference space, resulting in spatially normalised observations. The different acquisitions were analysed together, and an average bundle intensity map was obtained. The resulting distribution of bundles established within the stem is more representative than those obtained using a single image. The spatial normalisation procedure is generic and can be applied to other types of plants and other types of objects. In the present work, the projection onto a reference space used a rather simple polar transformation. In the case of more complicated shapes, other transformation procedures may be considered, including polynomial [23], thin-plate, or spline-based transformations [43]. Reference stems were built for each genotype. The model integrates the global stem morphology – stem size and shape – as well as the vascular bundle intensity. The intensity maps were estimated in the reference space common to all observations. One limit of this study is the lack of information for the region corresponding to the bark. While the bundle intensity in this region is high, its removal induces a bias in the estimation of the intensity map.

Vascular bundles were considered as points with a spatial distribution within the stem section. In the present work, the point process representing the vascular bundles was described by the local intensity estimated using classical kernel methods. Other estimation methods could be investigated, e.g., ones based on the distance to the *k*-th nearest neighbour [44], [45]. Bundle intensity maps will also be compared with other morphometric parameters, e.g., the average cell size or the local chemical composition [20]. Many tools have been developed within the framework of spatial statistics for the description and analysis of interactions between points of a process [16], [17]. Such tools make it possible to detect and quantify clustering or repulsion of the structures, allowing us to test hypotheses on the spatial organisation of bundles and to consider the modelling of the underlying point process. In particular, it may be possible to develop models that take both the heterogeneity within the section and the repulsion between bundles into account [46]. Cellular morphology is another key parameter for understanding the global properties of plant tissues [7], [8], [47]. The size and the shape of cells in a tissue have a direct impact on its mechanical properties and, hence, the mechanical behaviour of the plant or organ that contains it [48]. The description of cellular morphology is also of interest for studying the development of plant organs and understanding the mechanisms involved in plant morphogenesis [49], [50]. More generally, it is hoped to obtain a better description of the plant structure, histology and mechanical behaviour by coupling different models of the plant structure at various scales (organ, tissue, cell, cell wall, etc.) and using different modalities [19], [51]–[53].

## Supporting Information

File S1 Legend: Matlab and R scripts used to estimate groupwise bundle density maps.
(ZIP)

File S2 Data files containing bundle positions and slice contours.
(ZIP)

File S3 Source files of “Matgeom”, a library for geometric computing with Matlab, the Table class, and the ShapeViewer interface.
(ZIP)
