# Supplementary material for: Statistical Mapping of Maize Bundle Intensity at the Stem Scale Using Spatial Normalisation of Replicated Images
Source: PLoS One. 2014 Mar 12;9(3):e90673. doi: 10.1371/journal.pone.0090673 (PMC3951278; doi:10.1371/journal.pone.0090673)
Supplement: File S3 — Source files of “Matgeom”, a library for geometric computing with Matlab, the Table class, and the ShapeViewer interface. (ZIP) [file pone.0090673.s003.zip › libs/tableClass/demos/html/demoTable.html]

Using the Table Class 

# Using the Table Class

```
This is a demo file for presenting some features of the Table class.
```

```
 Usage:
   demoTable
```

## Contents

- Read and display data
- Plotting functions
- Management of groups
- Principal Component Analysis

## Read and display data

```
% Read data from a csv file (several options can be specified)
tab = Table.read('fisherIris.txt');

% display a part of the table on the console
disp(tab(1:5, :));

% Or display summary of the data, like in R
summary(tab);

% display the table in a frame
show(tab);
```

```
         SepalLength    SepalWidth    PetalLength    PetalWidth    Species
1                5.1           3.5            1.4           0.2     Setosa
2                4.9             3            1.4           0.2     Setosa
3                4.7           3.2            1.3           0.2     Setosa
4                4.6           3.1            1.5           0.2     Setosa
5                  5           3.6            1.4           0.2     Setosa


        SepalLength         SepalWidth        PetalLength         PetalWidth
    Min:     4.3000    Min:     2.0000    Min:     1.0000    Min:     0.1000
    1st Qu.: 5.1000    1st Qu.: 2.8000    1st Qu.: 1.6000    1st Qu.: 0.3000
    Median:  5.8000    Median:  3.0000    Median:  4.3000    Median:  1.3000
    Mean:    5.8433    Mean:    3.0540    Mean:    3.7587    Mean:    1.1987
    3rd Qu.: 6.4000    3rd Qu.: 3.3000    3rd Qu.: 5.1000    3rd Qu.: 1.8000
    Max:     7.9000    Max:     4.4000    Max:     6.9000    Max:     2.5000
                                                                            


           Species
    Setosa:     50
    Versicolor: 50
    Virginica:  50
```

## Plotting functions

```
% box plot of the quantitative variables
figure;
boxplot(tab(:,1:4));

% histogram of petal length. Columns can be indexed by their name.
figure;
hist(tab('PetalLength'));

% plot petal width against petal length
figure;
plot(tab('PetalLength'), tab('PetalWidth'), '*');
```

  

## Management of groups

```
% scatter plot using groups
figure;
scatterGroup(tab('PetalLength'), tab('PetalWidth'), tab('Species'), ...
    'Envelope', 'InertiaEllipse', ...
    'LegendLocation', 'NorthWest');

% Compute the mean of each group
disp(groupfun(tab(:,1:4), tab('Species'), @mean));
```

```
                          SepalLength    SepalWidth    PetalLength    PetalWidth
Species=Setosa                  5.006         3.418          1.464         0.244
Species=Versicolor              5.936          2.77           4.26         1.326
Species=Virginica               6.588         2.974          5.552         2.026
```

## Principal Component Analysis

```
% Apply Principal Component Analysis on the quantitative variables
irisPca = Pca(tab(:, 1:4), 'display', 'off');

% The result is a Pca object, containing Table object for scores, loadings
% and eigen values
disp(irisPca);

% Score plot can be displayed with automatic labeling of axes
figure;
scorePlot(irisPca, 1, 2);

% Loadings can also be displayed with automatic labeling
figure;
loadingPlot(irisPca, 1, 2);

% To display scores with group labelling, simply call the plot method on
% the score object stored in Pca result
figure;
scatterGroup(irisPca.scores(:, 1), irisPca.scores(:, 2), tab('Species'), ...
    'LegendLocation', 'NorthWest');
```

```
Principal Component Analysis Result
   Input data: fisherIris
       scaled: true
        means: <1x4 double>
       scores: <150x4 Table>
     loadings: <4x4 Table>
  eigenValues: <4x3 Table>
```

  

Published with MATLAB® 7.9
